# Parent-set bedtime in adolescence is associated with future cardiovascular disease risk: Evidence from the Add Health study

**DOI:** 10.1371/journal.pone.0339044

**Published:** 2025-12-16

**Authors:** Prince Nii Ossah Addo, Angela D. Liese, Jiajia Zhang, Glenn Weaver, Monique J. Brown

**Affiliations:** 1 Department of Epidemiology and Biostatistics, Arnold School of Public Health, University of South Carolina, Columbia, South Carolina, United States of America; 2 Department of Exercise Science, Arnold School of Public Health, University of South Carolina, Columbia, South Carolina, United States of America; 3 South Carolina SmartState Center for Healthcare Quality, Arnold School of Public Health, University of South Carolina, Columbia, South Carolina, United States of America; 4 Rural and Minority Health Research Center, Arnold School of Public Health, University of South Carolina, Columbia, South Carolina, United States of America; 5 Office for the Study on Aging, Arnold School of Public Health, University of South Carolina, Columbia, South Carolina, United States of America; Portugal Football School, Portuguese Football Federation, PORTUGAL

## Abstract

Parent-set bedtimes have been linked to a lower prevalence of key cardiovascular disease (CVD) risk factors in adolescents. However, little is known about how parent-set bedtimes during adolescence affect CVD risk later in life. This study examined the association between parent-set bedtimes and future CVD risk, as well as the potential mediating role of sleep health. Data were taken from Waves I and IV of the National Longitudinal Study of Adolescent to Adult Health, including 4,151 participants. Parent-set bedtimes (10:00 PM, by 11:00 PM, and by midnight) were collected at Wave I. The outcome measure was the 30-year Framingham CVD score, categorized as low or high risk. Analyses were performed using SURVEYLOGISTIC and CAUSALMED procedures in SAS. About 28% of adolescents had a parent-set bedtime by or after 11 PM, while 18% had no parent-set bedtime. Adolescents with parent-set bedtimes by or after midnight (aOR: 2.32, 95% CI: 1.65–3.26) and those without a parent-set bedtime (aOR: 1.35, 95% CI: 1.01–1.79) had significantly higher CVD risk in adulthood compared to those with earlier bedtimes (by 10 PM). Sleep health partially mediated the relationship between parent-set bedtime and future CVD risk. Our study findings indicate that parental-set bedtimes during adolescence are associated with future CVD risk. Further prospective or experimental studies are needed to confirm these relationships.

## Introduction

Adolescence is a significant stage of human development where physical, behavioral, and psychological changes that predict future health occur [1]. Behavioral patterns associated with diet, physical activity, and sleep developed during adolescence have beneficial or detrimental effects on health in adolescence and later life [2]. The American Academy of Sleep Medicine recommends that adolescents aged 13–17 years sleep 8–10 hours for optimal health [3]. However, adolescents face many challenges in getting enough sleep.

Compared to children, adolescents prefer late bedtimes (evening chronotype) because of changes in their biological clock [4–6]. When this preference for late bedtimes coincides with early school start times and other social demands, such as work or sports activities after school, it prevents them from getting enough sleep [7]. Further, the use of electronic devices around bedtime [8,9], poor sleep hygiene [8,10], mental health conditions [11], as well as neurodevelopmental disorders [12] have been shown to promote chronic sleep deprivation among adolescents.

Insufficient sleep among adolescents has been recognized as a public health problem in the United States (U.S.) [9]. This needs critical attention, since it negatively affects adolescents’ current and future health. The literature shows that sleep-deprived adolescents are at higher risk for metabolic issues, diabetes, and cardiovascular disease (CVD) in the future [13,14]. Sleep deprivation in adolescence has also been associated with increased cardiometabolic risk in adulthood [15,16]. Insufficient sleep in adolescence could therefore contribute significantly to the burden of CVD in the U.S., and the high economic and health costs associated with it [17]. Hence, interventions to improve sleep among adolescents could help their future cardiovascular health.

Research suggests that parent-set bedtimes offer some protection against depressive symptoms [18,19], later bedtimes [20,21], sleep deprivation [20,21], and help improve daytime functioning [21] among adolescents. Since sleep is a strong predictor of cardiovascular health, parent-set bedtime may offer some protection against CVD risk later in life. However, little is known about how parent-set bedtimes in adolescence influence CVD risk later in life. This study, therefore, examined the association between parent-set bedtimes and CVD risk later in life and the possible mediating role of sleep health in this association.

## Materials and methods

### Data source

This current study used data from the National Longitudinal Study of Adolescent to Adult Health (Add Health), a nationally representative sample of the U.S. adolescent population who were in grades 7–12 at the beginning of the study [22]. This cohort of adolescents has been followed through six waves of data collection, spanning from Wave I (1994–1995) to Wave VI (2022–2025). The Add Health study employs a stratified clustered sampling design and collects rich data on demographic factors, health-related behaviors, and physical and mental health through in-home interviews. At Wave I, parents of participating adolescents were also interviewed. Details of the Add Health study design and methods have been published elsewhere [22]. For this study, the Add Health public use dataset was accessed on April 3, 2023.

### Study sample

**Fig 1:**
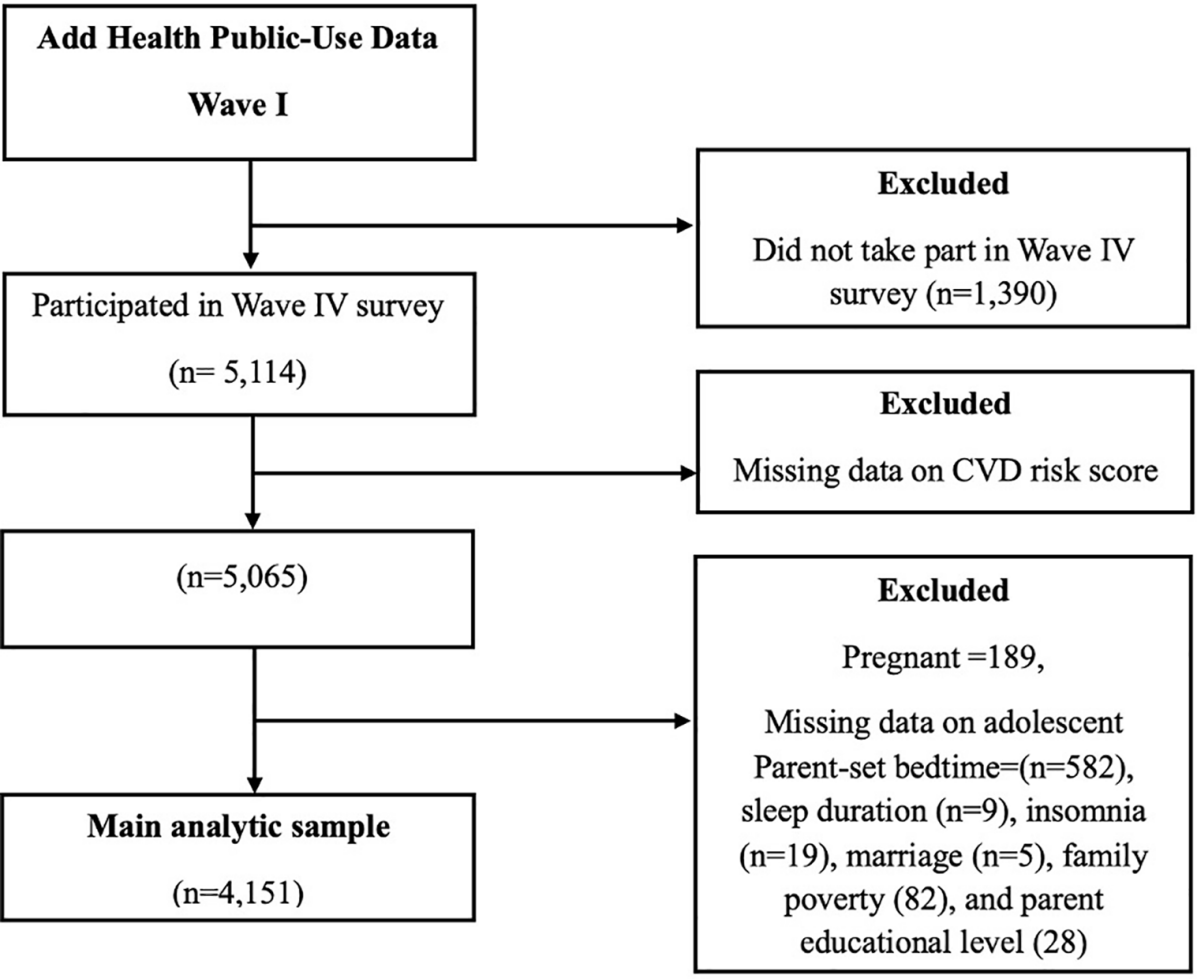
Flow chart showing the final analytic sample used in this study.

This current study used Waves I and IV of the Add Health public-use dataset. Participants included in this study were interviewed at Waves I and IV, were not pregnant, and had complete information on the main exposure, outcome, and confounding variables, as described in Fig 1. The final analytic sample used in this study comprised 4,151 participants. Participants included in this study were similar to those excluded in terms of demographics and main exposure factors but differed slightly by race/ethnicity (S1 Table). Written informed consent was provided by Add Health Study participants following the University of North Carolina School of Public Health Institutional Review Board (IRB) guidelines. Because this study used publicly available de-identified data, it was deemed exempt from IRB approval from the University of South Carolina Institutional Review Board.

## Measures

### Independent Variable: Parent-set bedtime

The primary independent variable in this study was the parent-set bedtime. Parents of adolescents who participated in Wave I in-home interviews were asked the following question: “*What time does {NAME} have to go to bed on weeknights?*” Responses included “9:00 PM or earlier, by 10:00 PM, by 11:00 PM; by 12:00 AM (midnight), by 1:00 AM, after 1:00 AM, (he/she) has no set bedtime.” Parent-set bedtime was categorized as “1=10:00 PM or earlier, 2=by 11:00 PM, 3=by or after midnight, and 4= no set bedtime” based on prior research [18].

### Dependent Variable: CVD risk score

CVD risk in adulthood was calculated using a prediction equation developed from the Framingham Heart Study [23]. This prediction equation estimates the risk of a CVD event (fatal or non-fatal stroke, myocardial infarction, congestive heart failure, angina pectoris, and coronary death) occurring within 30 years and accounts for competing causes of death. A 30-year CVD risk score was computed for each study participant based on self-reported (age, sex, smoking status, diabetes status, and use of hypertensive medication) and objectively measured [systolic blood pressure and body mass index (weight in kilograms/square of height in meters)] CVD risk factors assessed at wave IV. We used a SAS macro developed by research scientists from Kaiser Permanente [24] (https://github.com/zmn0322/30-Year-CVD-Risk-Prediction). Based on prior research, we categorized participants into low CVD risk (FRS <20%) and high CVD risk (FRS ≥ 20%), using a clinically relevant cutoff point of 20% [25,26].

### Mediator: Sleep health score

Sleep duration and insomnia were considered potential mediators of the association between parent-set bedtime and CVD risk in adulthood. Sleep duration was self-reported at Wave I and recategorized as “long (>12 hours for 6-12 years, >10 hours for 13-18 years, and >9 hours for 18-64 years), recommended (9-12 hours for 6-12 years, 8-10 hours for 13-18 years, and 7-9 hours for 18-64 years), and short (< 9 hours for 6-12 years, < 8 hours for 13-18 years, and < 7 hours for 18-64 years) [3]. Insomnia was measured with the following question: “In the past 12 months, how many times did you have trouble falling asleep or staying asleep?” Participants who reported trouble falling or staying asleep every day or almost every day in the past 12 months were recorded as having insomnia symptoms [27]. A sleep health score was computed based on sleep duration and insomnia. First, each sleep dimension was categorized as follows: “long and short sleep duration =1”, “recommended sleep duration =0”; “insomnia symptoms = 1”, and “no insomnia symptoms = 0”. A score was obtained by summing scores from these two sleep dimensions. Scores ranged from 0 to 2, with higher scores signifying poorer sleep health.

### Covariates

The following self-reported sociodemographic variables collected at Wave I were included as covariates due to their relationship with sleep and CVD: Age [16,28], sex [16,29], race/ethnicity [16,30], parent marital status, and socioeconomic status (measured using parent education and family poverty) [16,30]. Parents of participating adolescents were asked if they received social benefits such as unemployment (yes/no), supplemental security income (yes/no), food stamps (yes/no), social security (yes/no), or aid to families with dependent children (yes/no). A dichotomous family poverty variable was created from a positive response to any of the five questions above [31].

### Statistical analysis

Descriptive statistics were computed using survey procedures in SAS 9.4, which accounts for the clustered sampling design of the Add Health study and includes sampling weights. We calculated frequencies and percentages for categorical variables for the total analytic sample and by parent-set bedtime at Wave I. We used SURVEYLOGISTIC procedures in SAS to run crude and adjusted logistic regression analyses, controlling for the Add Health clustered sampling design by including sampling weights. We examined the association between parent-set bedtime and CVD risk in adulthood using parent-set bedtime of 10 PM or earlier as the reference group. We reported odds ratios with 95% confidence intervals.

We used the CAUSALMED procedure in SAS to examine the potential indirect (mediated) effect of sleep health on the association between parent-set bedtime and future CVD risk [32]. The CAUSALMED procedure estimates causal mediation effects within the counterfactual framework, allowing us to derive causal effects from observational studies. The method generates the total effect, direct effect, indirect (mediated) effect, and the percentage of the total effect that is attributed to mediation (percentage mediated) [32]. The exposure variable, parent-set bedtime, was dichotomized (no parent-set bedtime vs. 10 PM, 11 PM vs. 10 PM, and 12 AM vs. 10 PM) since the CAUSALMED procedure only works with continuous and binary exposures [32]. The covariates adjusted for in the primary analysis were also adjusted for in the mediation analysis. Responses of ‘don’t know’ or ‘refused’ were coded as missing responses.

**Fig 2:**
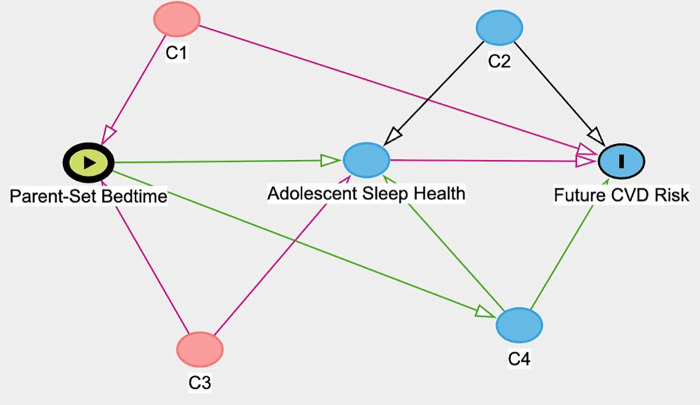
Directed acyclic graph of parent-set bedtime and future CVD risk showing mediating pathway (sleep health) and potential confounding pathways. C1: exposure-outcome confounder, C2: mediator-outcome confounder, C3: exposure-mediator confounder, and C4: mediator-outcome confounder caused by the exposure.

The directed acyclic graph (DAG) in Fig 2 illustrates the components of the mediation analyses for this study. Four assumptions must be met to estimate causal mediation effects: 1) no unmeasured confounding of the exposure-outcome relationship (C1); 2) no unmeasured confounding of the mediator-outcome relationship (C2); 3) no unmeasured confounding of the exposure-mediator relationship (C3); and 4) no mediator-outcome confounder that is caused by the exposure (C4). To evaluate the potential effect of unmeasured confounders on the associations, sensitivity analysis without assumptions (E-value) was conducted. The E-value represents the weakest association an unmeasured confounder must have with the exposure and outcome to provide an alternative explanation for the observed exposure-outcome association [33].

## Results

Table 1 presents the baseline characteristics of the study participants (n = 4,151). Sixty-eight percent of study participants were between the ages of 14 and 17 years, with a mean age of about 15 years. Most study participants were male (52.5%), non-Hispanic White (69.3%), had parents who were married (73.3%), and their families did not receive public assistance (77.3%). Approximately 38% of study participants slept fewer hours than the recommended amount for their age, and about one in ten participants reported insomnia symptoms at baseline (Table 1). Approximately 28% of adolescents had a parent-set bedtime by or after 11 PM, and around 18% had no parent-set bedtime. More than half of adolescents without a parent-set bedtime reported short sleep duration (53%) and usually went to bed at midnight or later (50%). Similarly, adolescents with parent-set bedtimes of midnight or later slept less than the recommended hours (53%) and usually went to bed after midnight (72%). About 20% fell within the high CVD risk category in adulthood.

**Table 1:** Adolescent background characteristics by parent-set bedtime (Wave I).

Characteristics	All	Parent-Set Bedtime				P-value
		None	By 10pm	By 11pm	By midnight	
**Unweighted (n)**	4,151	734	2,219	929	269	
**Total weighted %**	100	18.2	53.5	21.6	6.8	
**Age, years, *%***						**<.0001**
11-13	18.8	3.1	30.3	7.5	6.4	
14-17	68.0	66.3	64.3	79.5	65.5	
18-21	13.2	30.6	5.4	13.0	28.1	
**Sex, *%***						0.4568
Male	52.5	52.6	51.3	54.0	56.6	
Female	47.5	47.4	48.7	46.0	43.4	
**Race/Ethnicity, *%***						**0.0061**
Hispanic	10.1	6.3	11.7	9.1	11.7	
White NH	69.3	75.8	67.5	70.2	62.4	
Black NH	15.0	12.1	15.3	15.9	17.1	
Other NH	5.6	5.8	5.5	4.9	8.7	
**Parent Education, *%***						**<.0001**
Less than High School	14.9	10.6	17.6	11.8	15.2	
High school/GED	31.6	31.6	32.2	29.7	32.5	
Less than college degree	30.1	27.8	29.9	32.1	30.9	
College degree or above	23.4	29.9	20.2	26.4	21.5	
**Family Poverty, *%***						**0.0104**
Yes	22.7	17.8	24.6	21.3	25.5	
No	77.3	82.2	75.4	78.7	74.5	
**Parent Marital Status**						**0.0494**
Single/widowed	7.8	5.8	8.6	6.8	10.1	
Married	73.3	73.5	74.2	73.0	66.1	
Divorced/Separated	18.9	20.7	17.2	20.2	23.7	
^**a**^ **Sleep Duration**						**<.0001**
Recommended	59.4	44.9	69.7	51.4	42.0	
Short	37.6	53.0	27.1	45.7	53.1	
Long	3.0	2.1	3.2	2.8	4.8	
**Bedtime**						**<.0001**
10pm or earlier	41.7	17.7	63.8	17.9	7.9	
By 11pm	30.8	31.9	24.2	49.5	20.2	
By 12am	27.5	50.4	11.9	32.7	71.9	
**Adolescent-reported Insomnia**						0.1729
Yes	9.9	11.7	8.9	10.6	10.1	
No	90.1	88.3	91.1	89.4	89.9	
**Sleep Health Score**						**<.0001**
0	53.9	39.8	65.5	46.9	37.4	
1	40.6	53.6	29.9	47.0	57.1	
2	5.5	6.6	4.7	6.1	5.5	

^a^Sleep Duration: long (>12 hours for 6–12 years, > 10 hours for 13–18 year, and >9 hours for 18–64 years), recommended (9–12 hours for 6–12 years, 8–10 hours for 13–18 year, and 7–9 hours for 18–64 years), and short (< 9 hours for 6–12 years,<  8 hours for 13–18 years, and < 7 hours for 18–64 years)

### Bolded p-values denote statistical significance

Table 2 displays logistic regression results for CVD risk in adulthood. Adolescents with parent-set bedtimes of 11 PM (OR: 1.37, 95% CI: 1.12–1.68) and midnight (OR: 2.86, 95% CI: 2.01–4.05), and those with no parental set bedtime (OR: 1.67, 95% CI: 1.32–2.11), had increased odds of CVD compared to adolescents with earlier parent-set bedtimes (10 PM). After controlling for baseline demographic characteristics (age, sex, race/ethnicity, parent education, parent marital status, and family poverty), the odds ratios remained statistically significant, except for parent-set bedtime of 11 PM.

**Table 2:** Association between parent-set bedtime (Wave I) and CVD risk (Wave IV).

	High CVD risk vs. low CVD risk	
	Crude Model	^a^Adjusted Model
	OR (95% CI)	aOR (95% CI)
**Parent-set bedtimes**		
10 PM or earlier	1.00	1.00
By 11 PM	1.37 (1.12-1.68) **	1.18 (0.96-1.46)
By or after 12 AM (midnight)	2.86 (2.01-4.05) **	2.35 (1.69-3.29) **
No parent-set bedtime	1.67 (1.32-2.11) **	1.34 (1.01-1.77) *

CVD: cardiovascular disease, OR: odds ratio, CI: confidence interval

^a^Adjusted for age, sex, race/ethnicity, parent education, parent marital status, and family poverty at Wave I

*Indicates P-values ≤ 0.05; ** Indicates P-values ≤ 0.01

### Bolded odds ratios and 95% confidence intervals denote statistical significance

Table 3 presents the results of the causal mediation analysis. There were both direct and indirect effects of parent-set bedtime on future CVD risk. Adolescents with no parent-set bedtime had a significantly higher risk of CVD in adulthood (total effect OR: 1.38, 95% CI: 1.05–1.71), after adjusting for potential confounding factors. About 33% of the impact of not having a parent-set bedtime on future CVD risk could be attributed to sleep health (Table 3). However, sleep health did not mediate the relationship between a midnight parent-set bedtime and future CVD risk.

**Table 3:** Mediating role of sleep health in the causal effect of parent-set bedtimes on future CVD risk.

	OR (95% CI)	Proportion Mediated	E Value
^**a**^ **None (n=763) vs. 10PM (n=2,264)**			
Natural Direct effect	1.26 (0.95-1.56)		
Natural Indirect effect	1.10 (1.01-1.19) *		
Total effect	1.38 (1.05-1.71) *	32.66	1.63
**11PM (n=964) vs. 10PM (n=2,264)**			
Natural Direct effect	1.13 (0.89-1.38)		
Natural Indirect effect	1.06 (1.00-1.11)		
Total effect	1.20 (0.95-1.46)	32.83	
**12AM (n=269) vs. 10PM (n=2,264)**			
Natural Direct effect	2.02 (1.35-2.70) **		
Natural Indirect effect	1.05 (0.91-1.20)		
Total effect	2.13 (1.46-2.80) **	9.25	2.26

CVD: Cardiovascular disease, OR: odds ratio, CI: confidence interval

^a^None: no parent-set bedtime

*Indicates P-values ≤ 0.05; ** Indicates P-values ≤ 0.01

### Bolded odds ratios and 95% confidence intervals denote statistical significance

Models adjusted for age, sex, race/ethnicity, parent education, parent marital status, and family poverty at Wave I.

## Discussion

Building on prior research that has linked parent-set bedtimes to sleep health [20,21,34] and mental health [18,19] among adolescents, we sought to examine the possible long-term effects of parent-set bedtimes on adolescent future cardiovascular risk. In our study, adolescents with later parent-set bedtimes (by or after midnight) and those without a parent-set bedtime had a significantly higher CVD risk in adulthood, compared to adolescents with earlier parent-set bedtimes (by 10 PM). Sleep factors partially mediated the effect of parent-set bedtimes on future CVD risk.

In our study, earlier parent-set bedtimes were linked to a reduced future risk of CVD among adolescents. Several studies have shown a strong connection between parent-set bedtimes and established CVD risk factors such as poor-quality sleep [18,19,21] and depressive symptoms [18,19]. For example, a study by Short and colleagues found that adolescents with parent-set bedtimes had earlier bedtimes and more sleep on school nights, felt less tired, and experienced less daytime sleepiness compared with adolescents without parent-set bedtimes [21]. Other studies also report that earlier parent-set bedtimes are associated with earlier bedtimes, longer sleep duration, and a lower risk of suicide and depression, compared with late or nonexistent parent-set bedtimes [18,19].

Since sleep deprivation [16] and depression [35] in adolescence significantly increase CVD risk in adulthood, improving sleep quality and reducing depressive symptoms could have long-term beneficial effects on cardiovascular health, as evidenced by the results of our study. It is, however, essential to note that parent-set bedtimes have both direct and indirect effects on adolescent future CVD risk, as demonstrated through our mediation analysis. The impact of not having a parent-set bedtime in adolescence on future CVD risk was partially mediated by poor sleep health (higher sleep health score). However, the effect of having a parent-set bedtime of midnight in adolescence on future CVD risk was not mediated by sleep health; it was a direct effect. This indicates that a parent-set bedtime, in many ways, helps to improve sleep health among adolescents [20], which in turn may positively impact their cardiovascular risk in adulthood. On the other hand, not having a parent-set bedtime and parent-set bedtimes ‘by or after midnight’ may be detrimental to adolescents’ future cardiovascular health.

Interventions aimed at reducing CVD risk could therefore benefit greatly from parents setting and enforcing earlier bedtimes for their children. Recently published data suggest that even the reintroduction of bedtime rules in mid-adolescence has positive impacts on adolescent sleep health [20]. Public health experts, educators, and healthcare workers could therefore encourage parents to set and enforce bedtimes for their teenagers, as this may improve their sleep health and subsequent cardiovascular health.

## Strengths and limitations

To our knowledge, this is the first study to examine the influence of parent-set bedtimes in adolescence on future CVD risk. Additionally, this study expands the literature on the long-term impact of early life exposures, supporting the life course theory [36,37]. Further, the outcome measure used (30-year Framingham CVD risk score) was developed based on a relatively younger population, making it an appropriate risk score for the Add Health participants. Lastly, the findings of this study are generalizable to adolescents in the U.S., since the Add Health dataset is representative of this population, and sample weights were incorporated in the study.

However, the study findings must be interpreted with awareness of the following limitations. First, we were unable to examine important factors such as social jetlag, sleep regularity, and school start times, as well as potential confounders like family history of CVD and genetic predispositions, due to data constraints. Second, the primary exposure variable (parent-set bedtime) and mediator variable (sleep score) were self-reported, making them susceptible to measurement errors. Future research should consider incorporating objective sleep measures, such as actigraphy, to enhance understanding of sleep patterns. Lastly, since this study is observational, causality between parent-set bedtimes and cardiovascular outcomes cannot be established.

## Conclusion

Our study findings indicate that parental-set bedtimes during adolescence are associated with future CVD risk. Further prospective or experimental studies are needed to confirm these relationships.

## Supporting information

S1 Table Characteristics of included and excluded participants. (DOCX)
