# Supplementary material for: Parent-set bedtime in adolescence is associated with future cardiovascular disease risk: Evidence from the Add Health study
Source: PLoS One. 2025 Dec 16;20(12):e0339044. doi: 10.1371/journal.pone.0339044 (PMC12707677; doi:10.1371/journal.pone.0339044)
Supplement: S1 Table — (DOCX) [file pone.0339044.s001.docx]

**S1 Table. Characteristics of Participants Included and Excluded**

| **Characteristics** | **Included**  **(n=4,151)** | ***Excluded**  **(n=2,353)** |
| --- | --- | --- |
| **Age, years, *M (SD)*** | 15.37 (1.73) | 15.82 (1.84) |
| **Sleep Health Score, *M (SD)*** | 0.51 (0.60) | 0.52 (0.59) |
|  | **n (%)** | **n (%)** |
| **Sex** |  |  |
| Male | 1,991 (48.00) | 1,156 (49.15) |
| Female | 2,160 (52.00) | 1,196 (50.85) |
| **Race/Ethnicity** |  |  |
| Hispanic | 399 (9.61) | 344 (14.66) |
| Non-Hispanic White | 2,564 (61.77) | 1,172 (49.96) |
| Non-Hispanic Black | 946 (22.79) | 638 (27.20) |
| Other non-Hispanic | 242 (5.83) | 192 (8.18) |
| **Parent Education** |  |  |
| Less than High School | 582 (14.02) | 279 (19.08) |
| High school/GED | 1,244 (29.97) | 448 (30.64) |
| Less than college degree | 1,248 (30.07) | 422 (28.86) |
| College degree or above | 1,077 (25.95) | 313 (21.41) |
| **Family Poverty** |  |  |
| Yes | 944 (22.74) | 407 (27.86) |
| No | 3,207 (77.26) | 1,054 (72.14) |
| **Marital Status** |  |  |
| Single/widowed | 369 (8.89) | 172 (11.57) |
| Married | 2,980 (71.79) | 975 (65.57) |
| Divorced/Separated | 802 (19.32) | 340 (22.86) |
| **Sleep duration** |  |  |
| Recommended | 2,449 (59.00) | 1,336 (57.44) |
| Short | 1,590 (38.30) | 914 (39.29) |
| Long | 112 (2.70) | 76 (3.27) |
| **Insomnia** |  |  |
| Yes | 396 (9.54) | 225 (9.59) |
| No | 3,755 (90.46) | 2,120 (90.41) |
| **Parental Set Bedtime** |  |  |
| No set bedtime | 734 (17.68) | 252 (16.51) |
| By 10pm | 2,219 (53.46) | 801 (52.49) |
| By 11pm | 929 (22.38) | 369 (24.18) |
| By or after 12am | 269 (6.48) | 104 (6.82) |

*Percentages may not add up to 100 due to missing data
